# A Case of Kawasaki Disease with Coronary Aneurysm Responding to the 4th IVIG Treatment

**DOI:** 10.1155/2014/821812

**Published:** 2014-08-05

**Authors:** Ju Young Kim, Hyun Jung Kim

**Affiliations:** Department of Pediatrics, Eulji University Hospital, Eulji University School of Medicine, 1306, Dunsan-dong, Seo-gu, Daejeon 302-799, Republic of Korea

## Abstract

Kawasaki disease is an acute febrile illness that usually occurs in children younger than 5 years of age. The use of intravenous immunoglobulin (IVIG) within the first 10 days of illness has been shown to reduce the incidence of coronary artery aneurysms significantly. The relative roles of repeated doses of intravenous immunoglobulin (IVIG) are controversial in refractory Kawasaki disease (KD). Most experts recommend the second retreatment with IVIG, 2 g/kg in refractory KD. However, the dose-response effect of the third or fourth IVIG was uncertain. Although there have been a significant number of reports on new therapeutic options for refractory KD, such as steroid, infliximab, methotrexate, and other immunosuppressants, their effectiveness in reducing the prevalence of coronary artery aneurysms was unproven. We present here KD patient with small coronary artery aneurysm who is resistant to the third IVIG and steroid pulse therapy but showed improvement immediately after the infusion of the 4th IVIG on fever day 18.

## 1. Introduction

Kawasaki disease (KD) is an acute febrile, systemic vasculitic syndrome of unknown etiology, occurring primarily in children younger than 5 years of age. Administration of IVIG within the first 10 days after onset of fever in combination with high dose aspirin reduces the risk of coronary artery damage in KD [1]. IVIG treatment ≥10 days after illness onset was found to be insufficient for preventing coronary artery lesions [2]. IVIG should be administered to children presenting after the 10th day of illness if they have either persistent fever or aneurysms and ongoing systemic inflammation, as manifested by elevated erythrocyte sedimentation rate, C-reactive protein (CRP) [3]. However, the relative role of repeated doses of IVIG in refractory KD is still unknown. We report the case of refractory KD with coronary aneurysm responding the 4th IVIG treatment.

## 2. Case Description

**Figure 1:**
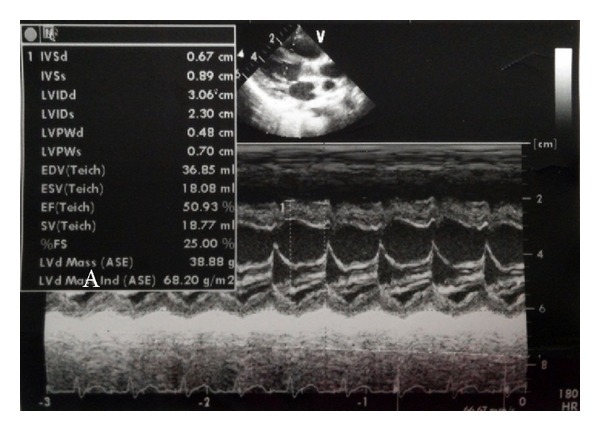
The echocardiographic findings on the second day of admission show decreased fractional shortening (25%) of left ventricle.

A 33-month-old boy presented with a 3-day history of fever and a 2-day history of skin rash, red eyes and lips, and increasing left neck pain. A physical examination upon admission revealed bilateral conjunctival injection without discharge, fissured red lips, strawberry tongue, and tender enlarged left cervical lymph nodes. The largest node, measuring 3 × 4 cm, was in the left posterior triangle of the neck. Breathing sounds were clear without crackles, and no cardiac murmur was audible. A chest radiograph was unremarkable, with a normal appearance of the mediastinum and no infiltrates. Initial laboratory results were as follows: haemoglobin 11.6 g/dL, white blood cell (WBC) count 25,730/*μ*L (polymorphonuclear leukocytes, 94.2%; lymphocytes, 1.9%; monocytes, 2.7%), platelet count 245 × 10^3^/*μ*L, CRP 28.5 mg/dL, sodium 130 mEq/L, aspartate aminotransferase (AST) 599 U/L, alanine aminotransferase (ALT) 288 U/L, total bilirubin 2.27 mg/dL, and N-terminal fragment of B-type natriuretic peptide (NT-proBNP) 4615 pg/mL. On hospital day 2, echocardiography revealed perivascular brightness around coronary arteries with decreased contractility of ventricle (Figure 1). The diagnosis of KD with acute myocarditis was made, and treatment with IVIG (Liv Gamma SN, SK) and high dose aspirin was begun. Intravenous cephalosporin was given concurrently due to elevated procalcitonin level (41 ng/mL). No clinical improvement occurred after IVIG treatment. The follow-up laboratory results on the third hospital day were as follows: haemoglobin 10.0 g/dL, WBC 10,010/*μ*L (polymorphonuclear leukocytes, 77.5%; lymphocytes, 14.1%; monocytes, 3.9%), platelets 262 × 10^3^/*μ*L, CRP 20.65 mg/dL, sodium 132 mEq/L, and the level of NT-proBNP was more elevated to 6212 pg/mL. On the 4th hospital day, the second dose of IVIG was infused. Because of the lack of improvement of the cervical adenopathy, neck computed tomography (CT) was performed to rule out a retropharyngeal abscess. Neck CT showed multiple enlarged lymph nodes in the left posterior cervical space, but no abscess-like lesion was observed.

Despite the infusion of the additional IVIG, the fever and other Kawaski features persisted. On the fifth hospital day, WBC 18,200/*μ*L (polymorphonuclear leukocytes, 77.5%; lymphocytes, 14.1%; monocytes, 3.9%), the total protein/albumin was 8.6/2.8 mg/dL, and the CRP level was elevated to 27.2 mg/dL. On the sixth hospital day, he was given the third IVIG infusion and oral dexamethasone (0.3 mg/kg/day) due to persistent fever and a repeat echocardiogram showing mild dilatation of the left main coronary artery (2.9 mm) and right coronary artery (2.7 mm). The fever subsided and his symptoms slowly resolved and the neck swelling continued to decrease until the ninth hospital day.

On hospital day 10, fever and other Kawasaki features (injected bulbar conjunctiva, fissured lips, and strawberry tongue) reappeared. He developed a productive cough. The follow-up laboratory results were as follows: haemoglobin 10.3 g/dL, WBC 23,730/*μ*L (polymorphonuclear leukocytes, 77.5%; lymphocytes, 14.1%; monocytes, 3.8%), platelets 628 × 10^3^/*μ*L, CRP 5.08 mg/dL, sodium 132 mEq/L, and positive mycoplasma pneumoniae IgM. The patient was given an additional dose of intravenous clarithromycin and pulse methylprednisolone (30 mg/kg/dose) on the 10th and 13th hospital days.

**Figure 2:**
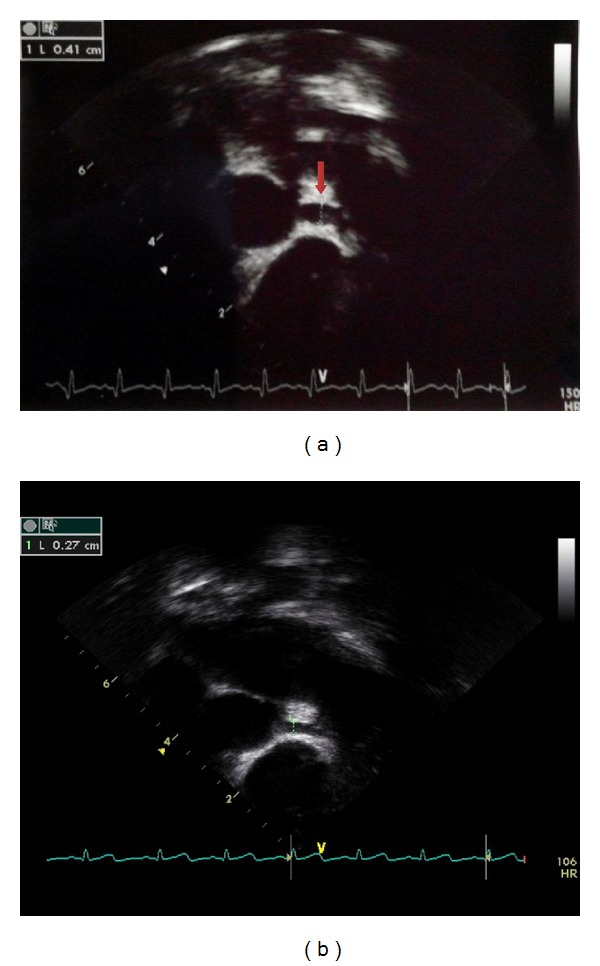
The echocardiographic findings on the 16th day of admission show fusiform aneurysm (4.1 mm) of left main coronary artery (a) but mild dilatation of left main coronary artery (2.7 mm) after the 4th IVIG  treatment (b).

On the 14th hospital day, he was in an afebrile state and was discharged on hospital day 16. On the night of discharge, he revealed bilateral conjunctival injection, fissured red lips, and edematous hands that led to being readmitted only a day after being discharged. There was more elevation of CRP (6.68 mg/dL) and platelets (998 × 10^3^/*μ*L) which suggested CAL. Echocardiogram (Figure 2) showed a small aneurysm of the left main coronary artery (4.1 mm) and mild dilatation of the left anterior descending coronary artery and right coronary artery (3.5 mm).

On hospital day 17, he was treated with the 4th IVIG (2 g/kg), aspirin (80 mg/kg/day), and clopidogrel (1 mg/kg/day). The following day, the fever eventually subsided and follow-up echocardiogram (Figure 2) showed mild dilatation of the left main coronary artery (2.7 mm) and a normal diameter of the left anterior descending coronary artery (2.1 mm), left circumflex artery (2.0 mm), and right coronary artery (2.3 mm). He has been under follow-up care for 6 months with no CAL.

## 3. Discussion

Intravenous immunoglobulin (IVIG) is pooled IgG from thousands of donors. Although administration of a combination of high dose IVIG and aspirin is the standard therapy for acute KD [1], 10–20% of patients with KD fail to defervesce with initial IVIG [4]. IVIG nonresponse is usually defined as persistent or recrudescent fever 36 hours or more after completion of 2 g/kg single infusion of IVIG [3]. IVIG nonresponders have a higher risk of coronary artery aneurysm (CAA), particularly giant and nongiant CAAs. A case-control study in Japan showed that the patients who received additional IVIG had a significantly higher risk for the development of giant CAAs although those patients were treated with various regimens of IVIG, not just the 2 g/kg single infusion of IVIG. Additional therapeutic trials for refractory KD have focused on resolution of the inflammation and prevention of coronary artery lesions (CAL) and have involved a second IVIG treatment, corticosteroids, immunosuppressants, and tumor necrosis factor-*α* (TNF-*α*) antagonists [5, 6]. Treatment strategies have varied between institutions, and guidelines for additional treatment have not yet been established [7].

The precise mechanism by which IVIG suppresses KD vasculitis is still unclear. Ramesh and other colleagues described the immunomodulatory action of IVIG [8]. It binds to the Fc receptor of B and T lymphocytes. This may inhibit antibody synthesis by B cells and/or alter regulatory T cell helper or suppressor activities. Recent data demonstrate that IVIG has direct inhibitory effects on leukocyte recruitment* in vitro* and* in vivo* through inhibition of selectin and integrin functions [9].

Most experts recommend retreatment with IVIG, 2 g/kg for those who fail to respond to initial therapy, although only two-thirds of these patients respond to the second IVIG [3]. The putative dose-response effect of IVIG forms the theoretical basis for this approach. However, the potential benefit of IVIG in refractory KD is more limited, in part because our understanding of the mechanisms underlying the effects of IVIG in the pathogenesis of KD is incomplete.

Studies of steroids as 2nd or 3rd line therapy for refractory KD are controversial. The results of a retrospective study suggested that administration of methylprednisolone pulse therapy (MPT) for IVIG-resistant KD may reduce the risk of CALs [10]. However, in a recent prospective study, MPT does not reduce the risk of development of CAL and does not seem to be beneficial as single agent therapy for IVIG resistant KD [11]. Our patient received two doses of MPT due to persistent fever after a third IVIG treatment. He became afebrile after receiving the MPT, but fever recurred 3 days later. And in this time, it was coincident with the dilatation of coronary arteries.

Circulating levels of TNF-*α* are markedly elevated during acute KD, and the degree of elevation correlates with the coronary artery damage and the development of aneurysms [12]. In current reports, 17 patients with acute KD were received infliximab infusion after at least two doses of IVIG and high dose aspirin [6]. In the above-mentioned cases, twelve patients had coronary artery abnormalities before infliximab therapy: four had transient dilatation that resolved after infliximab infusion, three had aneurysms, and five had ectasia. They showed that, with respect to the formation of CAL, infliximab treatment was superior to treatment with additional IVIG for KD that was intractable to the first IVIG. However, recent data show that infliximab was still detected 48 hours after infusion in the serum of a patient with disease refractory to infliximab, suggesting that unknown factors or cytokines other than TNF-*α* were responsible for the unresponsiveness and disease progression [13].

Back in our case, initially the patient had six principal symptoms and high risk factors of IVIG refractoriness including some of the laboratory parameters and the echocardiographic findings. He had a past history of diagnosis of KD at 27 months of age and complete recovery after the first IVIG and aspirin medication. This case leaves something to be desired because other intensive treatments besides IVIG may shorten the hospital length. Nevertheless, our case indicates that the more severe inflammation persists in a patient with KD, the more IVIG doses are required, and IVIG treatment ≥10 days after illness onset could be beneficial for preventing progression of CALs. It is remarkable that he had no coronary artery lesion eventually in spite of the long fever day in the acute stage of KD. However, it is controversial that the disappearance of coronary artery aneurysms is the direct effect of the 4th dose IVIG treatment. Because there are no consensus recommendations for the treatment of refractory KD, conduction of a large sized trials is needed to determine the role of repeated doses of IVIG according to the level of inflammation in patients with KD.
